# Male-specific association of the *FCGR2A* His167Arg polymorphism with Kawasaki disease

**DOI:** 10.1371/journal.pone.0184248

**Published:** 2017-09-08

**Authors:** Young-Chang Kwon, Jae-Jung Kim, Sin Weon Yun, Jeong Jin Yu, Kyung Lim Yoon, Kyung-Yil Lee, Hong-Ryang Kil, Gi Beom Kim, Myung-Ki Han, Min Seob Song, Hyoung Doo Lee, Kee-Soo Ha, Sejung Sohn, Ryota Ebata, Hiromichi Hamada, Hiroyuki Suzuki, Kaoru Ito, Yoshihiro Onouchi, Young Mi Hong, Gi Young Jang, Jong-Keuk Lee, the Korean Kawasaki Disease Genetics Consortium

**Affiliations:** 1 Asan Institute for Life Sciences, University of Ulsan College of Medicine, Seoul, Korea; 2 Department of Pediatrics, Chung-Ang University Hospital, Seoul, Korea; 3 Department of Pediatrics, University of Ulsan College of Medicine, Asan Medical Center, Seoul, Korea; 4 Department of Pediatrics, Kyung Hee University Hospital at Gangdong, Seoul, Korea; 5 Department of Pediatrics, The Catholic University of Korea, Daejeon St. Mary’s Hospital, Daejeon, Korea; 6 Department of Pediatrics, Chungnam National University Hospital, Daejeon, Korea; 7 Department of Pediatrics, Seoul National University Children's Hospital, Seoul, Korea; 8 Department of Pediatrics, University of Ulsan, Gangneung Asan Hospital, Gangneung, Korea; 9 Department of Pediatrics, Inje University Haeundae Paik Hospital, Busan, Korea; 10 Department of Pediatrics, Pusan National University Hospital, Busan, Korea; 11 Department of Pediatrics, Korea University Hospital, Seoul, Korea; 12 Department of Pediatrics, Ewha Womans University Hospital, Seoul, Korea; 13 Department of Pediatrics, Chiba-University Graduate School of Medicine, Chiba, Japan; 14 Department of Pediatrics, Tokyo Women’s Medical University Yachiyo Medical Center, Yachiyo, Japan; 15 Department of Pediatrics, Wakayama Medical University, Wakayama, Japan; 16 Laboratory for Cardiovascular diseases, RIKEN Center for Integrative Medical Sciences, Yokohama, Japan; 17 Department of Public Health, Chiba University Graduate School of Medicine, Chiba, Japan; Kaohsiung Chang Gung Memorial Hospital, TAIWAN

## Abstract

Kawasaki disease (KD) is an acute systemic vasculitis that can potentially cause coronary artery aneurysms in some children. KD occurs approximately 1.5 times more frequently in males than in females. To identify sex-specific genetic variants that are involved in KD pathogenesis in children, we performed a sex-stratified genome-wide association study (GWAS), using the Illumina HumanOmni1-Quad BeadChip data (249 cases and 1,000 controls) and a replication study for the 34 sex-specific candidate SNPs in an independent sample set (671 cases and 3,553 controls). Male-specific associations were detected in three common variants: rs1801274 in *FCGR2A* [odds ratio (OR) = 1.40, *P* = 9.31 × 10^−5^], rs12516652 in *SEMA6A* (OR = 1.87, *P* = 3.12 × 10^−4^), and rs5771303 near *IL17REL* (OR = 1.57, *P* = 2.53 × 10^−5^). The male-specific association of *FCGR2A*, but not *SEMA6A* and *IL17REL*, was also replicated in a Japanese population (OR = 1.74, *P* = 1.04 × 10^−4^ in males vs. OR = 1.22, *P* = 0.191 in females). In a meta-analysis with 1,461 cases and 5,302 controls, a very strong association of KD with the nonsynonymous SNP rs1801274 (p.His167Arg, previously assigned as p.His131Arg) in *FCGR2A* was confirmed in males (OR = 1.48, *P* = 1.43 × 10^−7^), but not in the females (OR = 1.17, *P* = 0.055). The present study demonstrates that p.His167Arg, a KD-associated *FCGR2A* variant, acts as a susceptibility gene in males only. Overall, the gender differences associated with *FCGR2A* in KD provide a new insight into KD susceptibility.

## Introduction

Recent studies have shown that sex-related differences in the genetic architecture may contribute to complex diseases that are more prevalent in one sex over the other in the general population [1]. Autoimmune diseases, such as systemic lupus erythematosus, Sjogren's syndrome, and rheumatoid arthritis, occur more frequently in women than in men [2]. However, certain types of autoimmune diseases, such as Goodpasture syndrome in which antibodies attack the basement membrane in lung and kidney cells, occur more commonly in men than in women [3]. In addition, there is growing evidence that sex hormones may be attributed to the sex-related differences in humoral and cellular responses as well as in infections and vaccinations [4,5]. For example, males were predominantly more susceptible to many infectious diseases than females, and antibody responses against viral vaccines were higher in females than in males [6–9]. However, although there are differences in the incidence of many infectious or autoimmune diseases by gender, the mechanism remains unknown.

KD is a self-limited vasculitis mainly affecting children under 5 years of age [10,11]. Although cases of KD have been reported in all ethnic origins, KD is more common in children of Asian descent [12]. KD commonly affects boys 1.5 times more than girls [13]. The epidemiological data on the sex ratio in KD is often overlooked in understanding the pathophysiological mechanisms of KD. Furthermore, genome-wide association studies (GWASs) have identified *FCGR2A*, *BLK*, *CD40*, and the human leukocyte antigen (HLA) class II region as KD susceptibility loci [14–16]. However, any sex-specific genetic factors in KD have not yet been identified. To identify sex-specific susceptibility genes of KD, we performed a sex-stratified GWAS analysis and replication study. We found that the *FCGR2A*, *SEMA6A*, and *IL17REL* loci were significantly associated with KD in males but not in females in the Korean population. Male-specific association of the functional polymorphism (rs1801274; p.His167Arg, previously assigned as p.His131Arg) of *FCGR2A* with KD was also replicated in the Japanese population.

## Materials and methods

### Study subjects and genotype data

All of our patients with KD were diagnosed by pediatricians according to the diagnostic criteria of the American Heart Association [17]. Information detailing our samples has been described in our previous works [18,19]. A total of 249 patients with KD and 4,553 control subjects were genotyped on the Illumina HumanOmni1-Quad BeadChip for a GWAS. Additional genotyping of 666 case samples for replication study was performed on the high-throughput Fluidigm EP1 system (Fluidigm Corp., South San Francisco, CA) using the Fluidigm SNP Type^™^ assay platform according to the manufacturer’s instructions. A total of 4,553 control subjects, including the 1,000 control subjects (783 males and 217 females) used in the initial GWAS and the 3,553 control subjects (1,079 males and 2,474 females) used in the replication study, were included from the adult health cohort of the general population in Korea. The genotypes of the control subjects were provided by the Biobank for Health Sciences at the Center for Genome Sciences in Chungwon, Korea. Our BeadChip data, including 249 KD cases, were also deposited to the Biobank of Health Sciences (http://koreabiobank.re.kr; accession number: 2014-ER7402-00). A replication study was also performed in a Japanese population. The genotype data for the Japanese population was obtained using Invader assay in 546 patients with KD (320 males and 226 females) and 749 control subjects (405 males and 344 females) recruited from several medical institutes in Japan. All Japanese patients were diagnosed as having KD by pediatricians according to previously published diagnostic guideline [20]. The Institutional Review Boards of RIKEN and the other medical institutes in Japan approved the study and all of the patients’ parents and healthy volunteers gave written informed consent. To find and compare the coding variants of three male-associated susceptibility genes for KD (*FCGR2A*, *SEMA6A*, and *IL17REL*), we searched our in-house whole exome sequencing data that included a total of 100 cases of KD and 247 control samples. Written informed consents were obtained from the parents of the patients with KD, in accordance with our Institutional Review Boards (IRB). This study was approved by the IRB at Asan Medical Center (S2014-1339-0011).

### Statistical analysis

The genetic associations of the single nucleotide polymorphisms (SNPs), including the meta-analyses, were analyzed using PLINK (ver 1.07) [21]. A chi-square test was performed to compare allelic frequencies between the cases and the controls. Genetic associations were estimated by the odds ratios (OR) with 95% confidence intervals (CI). From an initial sex-stratified GWAS analysis (case-male vs. control-male and case-female vs. control-female), a total of 39 SNPs and 44 SNPs were chosen as male-specific and female-specific KD susceptibility loci, respectively. Finally, we narrowed down to 18 male-specific and 16 female-specific candidate SNPs for a replication study by selecting loci that were related to immune responses and sex-specific genetic associations. In a meta-analysis, between-study heterogeneity was assessed by calculating the Q-statistic (statistically significant if *P* < 0.05) [22] and quantified using the I^2^ value (statistically significant if I^2^ > 50%) that describes the percentage of variation across studies that are due to heterogeneity rather than chance [23]. The pooled ORs were calculated using the fixed or random-effect model [24,25]. To evaluate the significance of differences in the distribution of variables of clinical characteristics in each genotype group, all analyses were conducted using the moonBook package [26] within the R statistical language [27]. A Shapiro-Wilk test was performed for a normality test of the continuous variables. The continuous variables in our lab data with a non-normal distribution were described by median and interquartile range (IQR). The difference between the genotype groups was tested by the Fisher’s exact test for the categorical variables and by the Kruskal-Wallis rank sum test for the interval scale measurements.

## Results

### Identification of *IL17REL*, *FCGR2A*, and *SEMA6A* as male-specific KD susceptibility loci in the Korean population

To identify sex-specific genetic variants associated with KD, we carried out sex-stratified GWAS, using data from 249 cases (165 males and 84 females) and 1,000 control subjects (783 males and 217 females) that were genotyped with the Illumina Omni1-Quad BeadChip. A total of 34 sex-specific candidate SNPs that are associated with KD were chosen for a replication study in an independent sample set consisting of 666 patients with KD (385 males and 281 females) and 3,553 control subjects (1,079 males and 2,474 females). We observed three SNPs that were male-specific: rs5771303 near *IL17REL* (OR = 1.48, *P* = 0.00233 in males vs. OR = 1.02, *P* = 0.867 in females), rs1801274 (p.His167Arg) in *FCGR2A* (OR = 1.29, *P* = 0.0136 in males vs. OR = 1.14, *P* = 0.235 in females), and rs12516652 (p.Asp567Glu) in *SEMA6A* (OR = 1.58, *P* = 0.0357 in males vs. OR = 1.30, *P* = 0.231 in females) (Table 1). In a combined analysis, rs5771303 near *IL17REL* was the SNP most significantly associated with KD in males (OR = 1.57, *P* = 2.53 × 10^−5^), but not in females (OR = 1.04, *P* = 0.730). Furthermore, rs1801274 (p.His167Arg) in *FCGR2A* was also specifically associated with KD in males (OR = 1.40, *P* = 9.31 × 10^−5^), but not in females (OR = 1.16, *P* = 0.121). We also found an association between rs12516652 (p.Asp567Glu) in *SEMA6A* and KD in males (OR = 1.87, *P* = 3.12 × 10^−4^), but not in females (OR = 1.10, *P* = 0.642) (Table 1). Furthermore, in our whole exome sequencing data (100 KD cases vs. 247 controls), we found that the same nonsynonymous SNP (rs12516652) is significantly associated with KD in males (OR = 6.07, *P* = 7.54 × 10^−5^), but not in females (OR = 0.21, *P* = 0.116) (S1 Table).

**Table 1:** Male-specific association of rs1801274 in *FCGR2A*, rs12516652 in *SEMA6A*, and rs5771303 near *IL17REL* with KD.

SNP	Cytomap	Locus	Risk allele	Collection	Males				Females			
					No. (case/control)	RAF (case/control)	OR (95% CI)	*P*	No. (case/control)	RAF (case/control)	OR (95% CI)	*P*
rs1801274	1q23	*FCGR2A*	A	Korea GWAS	165/783	0.835/0.752	1.67 (1.22–2.29)	**1.21 × 10**^**−3**^	84/217	0.800/0.767	1.21 (0.78–1.88)	0.385
				Korea replication	385/1,079	0.805/0.762	1.29 (1.05–1.58)	**1.36 × 10**^**−2**^	281/2,474	0.783/0.760	1.14 (0.92–1.40)	0.235
				Korea combined	550/1,862	0.814/0.758	1.40 (1.18–1.66)	**9.31 × 10**^**−5**^	365/2,791	0.787/0.761	1.16 (0.96–1.40)	0.121
				Japan replication	320/405	0.864/0.785	1.74 (1.31–2.30)	**1.04 × 10**^**−4**^	226/344	0.823/0.792	1.22 (0.90–1.66)	0.191
				Meta-analysis	870/2,267		1.48 (1.28–1.71)	**1.43 × 10**^**−7**^	591/3,035		1.17 (1.00–1.37)	0.055
rs12516652	5q23	*SEMA6A*	A	Korea GWAS	165/783	0.056/0.023	2.53 (1.42–4.52)	**1.15 × 10**^**−3**^	84/217	0.018/0.039	0.45 (0.15–1.54)	0.191
				Korea replication	385/1,079	0.044/0.028	1.58 (1.03–2.42)	**3.57 × 10**^**−2**^	281/2,474	0.044/0.035	1.30 (0.85–2.00)	0.231
				Korea combined	550/1,862	0.048/0.026	1.87 (1.32–2.63)	**3.12 × 10**^**−4**^	365/2,791	0.038/0.035	1.10 (0.73–1.65)	0.642
				Japan replication	320/405	0.028/0.021	1.35 (0.69–2.64)	0.379	226/344	0.022/0.035	0.63 (0.30–1.32)	0.215
				Meta-analysis	870/2,267		1.74 (1.28–2.36)	**3.79 × 10**^**−4**^	591/3,035		1.01 (0.62–1.65)	0.968
rs5771303	22q13	*IL17REL*	C	Korea GWAS	165/783	0.899/0.837	1.75 (1.19–2.56)	**3.99 × 10**^**−3**^	84/217	0.859/0.825	1.29 (0.78–2.12)	0.313
				Korea replication	385/1,079	0.890/0.845	1.48 (1.15–1.91)	**2.33 × 10**^**−3**^	281/2,474	0.861/0.859	1.02 (0.79–1.31)	0.867
				Korea combined	550/1,862	0.893/0.841	1.57 (1.27–1.93)	**2.53 × 10**^**−5**^	365/2,791	0.861/0.856	1.04 (0.83–1.30)	0.730
				Japan replication	320/405	0.789/0.792	0.98 (0.76–1.27)	0.900	226/344	0.821/0.778	1.31 (0.97–1.77)	0.083
				Meta-analysis	870/2,267		1.29 (1.10–1.52)	**2.10 × 10**^**−3**^	591/3,035		1.15 (0.96–1.38)	0.129

A meta-analysis was performed using 3 collections (Korean genome-wide association study [GWAS], Korean replication study, and Japanese replication study) with 1,461 cases of KD (870 males and 591 females) and 5,302 control subjects (2,267 males and 3,035 females)

These statistical values are for the allelic model and significant *P*-values (*P* <0.05) are shown in bold.

KD, Kawasaki disease; SNP, single nucleotide polymorphism; No., number; RAF, risk allele frequency; OR, odds ratio; 95% CI, 95% confidence interval.

### Validation of *FCGR2A*, but not *SEMA6A* and *IL17REL*, as male-specific KD susceptibility gene in the Japanese population

To further validate our findings in other populations, we performed a replication study with a Japanese population of 546 Japanese patients with KD (320 males and 226 females) and 749 Japanese control subjects (405 males and 344 Females). In a Japanese sex-stratified association analysis, no significant associations were observed for rs12516652 in *SEMA6A* in either the male or female group (*P* = 0.379 in males and *P* = 0.215 in females) (Table 1). Furthermore, no significant association was observed for rs5771303 near *IL17REL* in either the male or female group as well *(P* = 0.900 in males and in *P* = 0.083 females) (Table 1). However, rs1801274 (p.His167Arg) in *FCGR2A* was predominately associated with KD susceptibility in males (OR = 1.74, *P* = 1.04 × 10^−4^), but not in females (OR = 1.22, *P* = 0.191). In a meta-analysis comprising 1,461 patients (870 males and 591 females) and 5,302 control subjects (2,267 males and 3,035 females), we found a strong association between rs1801274 (p.His167Arg) and KD in males (OR = 1.48, *P* = 1.43 × 10^−7^), but not in females (OR = 1.17, *P* = 0.055) (Table 1), indicating that *FCGR2A* influences KD susceptibility in male patients only. In addition, when we analyzed our clinical data for *FCGR2A* SNP rs1801274 in either male (n = 550) or female (n = 365) patients, we observed a significant gender-related difference in the median age of patients with KD, showing that male patients with KD with risk allele A of rs1801274 were younger than non-risk homozygotes (AA = 2.5 years, CA = 2.6 years, CC = 3.9 years, P = 0.013), compared to female patients (AA = 2.6 years, CA = 2.8 years, CC = 3.2 years, P = 0.636) (S2 Table). This result indicates that the risk allele A of *FCGR2A* results in high susceptibility and subsequent development of KD at an early age.

## Discussion

Although a difference in the prevalence of KD between male and female patients have been previously observed [11,28] the genetic contributions for these gender-specific differences have not yet been clarified. Here, we investigated the genetic effects of gender on KD susceptibility. Through a sex-stratified GWAS and a replication study, we found that the *IL17REL*, *FCGR2A*, and *SEMA6A* loci are associated with KD in males but not in females in the Korean population. Furthermore, the *FCGR2A* locus, but not the *IL17REL* and *SEMA6A* loci, also showed a male-specific association with KD in a Japanese population.

Two of the SNPs associated with males in Korean population are nonsynonymous coding variants including rs1801274 in *FCGR2A* (c.519A<G; p.His167Arg, which was previously assigned as His131Arg) and rs12516652 in *SEMA6A* (c.1701C>A; p.Asp567Glu). Although a functional SNP (rs1801274) and methylation sites of *FCGR2A* has been previously reported to be associated with KD [14,29–31] and other autoimmune diseases [32], these studies did not consider gender differences and their role in KD. *FCGR2A* encodes a low-affinity receptor for the constant fragment of immunoglobulin (Ig) G that is expressed on neutrophils, monocytes, and macrophages. FCGR2A transduces an activation signal for phagocytosis and the release of inflammatory mediators when ligated with immune complexes [33,34]. The rs1801274 (p.His167Arg) in *FCGR2A* has also been reported to be at the contact interface between the FCGR2A receptor and IgG, therefore affecting the affinity of FCGR2A for IgG subclasses [35]. The His131 allelic variant of *FCGR2A* was reported to readily bind IgG2, whereas the Arg131 allelic variant does not bind IgG2 as effectively [36,37]. In addition, the His131 allelic variant may bind IgG3 with moderately greater affinity than the Arg131 allelic variant [37,38]. Therefore, we hypothesize that the His131 allelic variant has increased binding affinity to the IgG subclasses and enhances the immune response to infectious agents, overproduction of cytokines, and activation of B cells. Although a sex-specific immunological role of *FCGR2A* has not yet been investigated, rs1801274 (p.His167Arg) has been reported to be associated with susceptibility to malaria infections during pregnancy, owing to hormonal fluctuations [39]. Our results further suggest that the His167 allelic variant might play a crucial role in the pathogenesis of KD in males but not in females.

Additionally, *SEMA6A* is located on chromosome 5q23 and encodes a member of the semaphorin family that consists of a Sema functional domain and an integrin-like extracellular domain that determines binding specificity. *SEMA6A* plays an important role in the development of the neural tissue, regulation of cell adhesion and motility, angiogenesis, the immune responses, and tumorigenesis [40–42]. In our GWAS and a replication study, we found that rs12516652, a nonsynonymous SNP in *SEMA6A* (c.1701C>A; p.Asp567Glu) is significantly associated with KD in males (OR = 1.87, *P* = 3.12 × 10^−4^), but not in females (OR = 1.10, *P* = 0.642) in the Korean population (Table 1). In whole exome sequencing data, we also found that the same SNP (rs12516652) is significantly associated with KD in males (OR = 6.07, *P* = 7.54 × 10^−5^), but not in females (OR = 0.21, *P* = 0.116) (S1 Table). Intriguingly, although the mechanism for the male-specific association of *SEMA6A* with KD remains unknown, the *SEMA6A* locus has been reported to be a significant contributor to risk for granulomatosis with polyangiitis, which is a systemic vasculitis characterized by vessel inflammation and granuloma formation affecting the respiratory tract, kidneys, and other organs and tissues [43]. This result suggests that both KD and granulomatosis with polyangiitis may share the *SEMA6A* variant as a genetic risk factor and its subsequent male predominance of incidence.

Finally, *IL17REL* is located on chromosome 22q13, and its extracellular receptor domains share homology with *IL17RE* and other members of the *IL17* receptor family. Furthermore, these extracellular receptor domains oligomerize to form functional receptor complexes and functions in the *IL17* pathway to initiate the T_H_2-mediated immune response [44,45]. Although *IL17RE* has been reported to be a functional receptor for *IL17C* as well as mediating the mucosal immunity in the small intestine of mice during acute experimental colitis [46], the overall function of *IL17REL* still remains unclear. However, several nonsynonymous SNPs in *IL17REL* have been demonstrated to be associated with ulcerative colitis (p.Leu333Pro, rs5771069; p.Pro262Leu, rs142430606; p.Glu151Gly, rs200958270) [47,48] and atrial fibrillation (p.Glu151Gly, rs200958270), the latter of which is the most common cardiac arrhythmia in males [49–51]. These studies suggest an intriguing possibility linking the *IL17REL* variants with a male predominance of the incidence of KD and other human diseases. On the other hand, we found that rs5771303, the most significantly associated male-specific SNP near the *IL17REL* locus, was located approximately 10 kb upstream of *IL17REL*. We were also unable to identify any additional significantly associated coding SNPs in *IL17REL* through our whole exome sequencing data analysis (S1 Table). Interestingly, *IL17REL* gene expression was only detected in the male gonads and spleen (https://www.ncbi.nlm.nih.gov/UniGene/ESTProfileViewer.cgi?uglist=Hs.526712). These observations suggest that the regulation of *IL17REL* expression may play an important role in a male-specific susceptibility to KD.

Our study is the first GWAS to identify sex-specific susceptibility genes in KD. The male-specific KD susceptibility of *FCGR2A* was validated in both Korean and Japanese population. However, the *SEMA6A* and *IL17REL* were not replicated in the Japanese population. The inconsistency may be due to the small sample size of the replication study in the Japanese population or population heterogeneity since we found significant heterogeneity of the *IL17REL* locus between Korean and Japanese population (S3 Table). Therefore, the male-specific association of *SEMA6A* and *IL17REL* is required for further validation. It is also necessary to validate a functional role for *IL17REL* and *SEMA6A* for sex-specific effects on KD susceptibility.

In conclusion, we identified rs1801274 (p.Arg167His; previously assigned as p.Arg131His), a nonsynonymous SNP in *FCGR2A*, to be significantly associated with KD in males but not in females. These findings might provide new insights into the predominance of KD incidence in male patients.

## Supporting information

S1 Table Sex-specific association of KD with nonsynonymous SNPs found in *FCGR2A*, *SEMA6A*, and *IL17REL* from whole exome sequencing data (100 KD cases vs. 247 controls). These statistical values are for the allelic model and significant *P*-values (*P* <0.05) are shown in bold.
(DOCX)

S2 Table Clinical characteristics of patients with KD by gender and rs1801274 (*FCGR2A*, risk allele: A) genotypes. The results for the categorical variables are presented in percentages in round brackets for the three genotype groups. For the normality test of the continuous variables, the Shapiro-Wilk test was used. The continuous variables were found to be non-normally distributed and described by median and interquartile range (IQR) in squared brackets. The difference between the groups was tested by Fisher’s exact test for the categorical variables and by the Kruskal-Wallis rank sum test for the interval scale measurements. A *P*-value of < 0.05 was considered as statistically significant. *Variables (diameter of coronary artery at worst and neutrophil %) tested for statistical significance by linear regression with age as a covariate. Significant *P*-values (*P* <0.05) are shown in bold.
(DOCX)

S3 Table Between-study heterogeneity in a meta-analysis of associations of rs1801274 in *FCGR2A*, rs12516652 in *SEMA6A*, and rs5771303 near *IL17REL* with KD. A meta-analysis was performed using 3 collections including Korea GWAS, Korea replication and Japan replication data. These *P*-values are for the allelic model. Pooled ORs were calculated by the fixed or random-effect model. The Cochran’s Q-statistic (*P*_het_) and I^2^ test were used to evaluate the between-study heterogeneity. If a *Q*-test showed a *P*_het_ < 0.05 or an I^*2*^ test exhibited > 50%, each of which indicates significant heterogeneity (marked in bold).
(DOCX)

S1 Dataset Genotype data and clinical data used in this study were provided in excel file. (XLSX)

## Abbreviations

CI: Confidence interval
GWAS: Genome-wide association study
HLA: Human leukocyte antigen
IQR: Interquartile range
IRB: Institutional review board
KD: Kawasaki disease
OR: Odds ratio
SNP: Single nucleotide polymorphism
