# Supplementary material for: Male-specific association of the FCGR2A His167Arg polymorphism with Kawasaki disease
Source: PLoS One. 2017 Sep 8;12(9):e0184248. doi: 10.1371/journal.pone.0184248 (PMC5590908; doi:10.1371/journal.pone.0184248)
Supplement: S1 Table — These statistical values are for the allelic model and significant P-values (P <0.05) are shown in bold. (DOCX) [file pone.0184248.s001.docx]

**S1 Table. Sex-specific association of KD with nonsynonymous SNPs found in *FCGR2A*, *SEMA6A,* and *IL17REL* from whole exome sequencing data (100 KD cases vs. 247 controls)**

| Locus | SNP | Allele | Amino acid | Males  (64 KD vs. 180 Controls) | | |  | Females  (36 KD vs. 67 Controls) | | |
| --- | --- | --- | --- | --- | --- | --- | --- | --- | --- | --- |
|  |  |  |  | RAF (case/control) | OR (95% CI) | *P* |  | RAF (case/control) | OR (95% CI) | *P* |
| *FCGR2A* | rs9427397 | C>T | Gln63Ter | 0.0/0.0 | na | na |  | 0.014/0.0 | na | 0.172 |
|  | rs9427398 | A>G | Gln63Arg | 0.0/0.0 | na | na |  | 0.014/0.0 | na | 0.172 |
|  | rs4986941 | A>G | Met140Val | 0.008/0.0 | na | 0.093 |  | 0.0/0.0 | na | na |
|  | rs1801274 | A>G | His167Arg | 0.836/0.764 | 1.56 (0.93−2.70) | 0.090 |  | 0.778/0.806 | 0.84 (0.42−1.69) | 0.632 |
|  | rs382627 | T>C | Leu274Pro | 0.016/0.028 | 0.55 (0.12−2.57) | 0.446 |  | 0.042/0.037 | 1.12 (0.26−4.83) | 0.878 |
|  |  |  |  |  |  |  |  |  |  |  |
| *SEMA6A* | rs12516652 | C>A | Asp567Glu | 0.094/0.017 | 6.07 (2.23−16.5) | **7.54 × 10^-5^** |  | 0.014/0.062 | 0.21 (0.03−1.75) | 0.116 |
|  |  |  |  |  |  |  |  |  |  |  |
| *IL17REL* | rs9617090 | G>A | Gly79Arg | 0.328/0.330 | 0.99 (0.64−1.52) | 0.9617 |  | 0.375/0.299 | 1.41 (0.77−2.58) | 0.264 |

These statistical values are for the allelic model and significant *P*-values (*P* <0.05) are shown in bold.

KD, Kawasaki disease; SNP, single nucleotide polymorphism; RAF, risk allele frequency; OR, odds ratio; 95% CI, 95% confidence interval; na, not available.
