# Supplementary material for: Male-specific association of the FCGR2A His167Arg polymorphism with Kawasaki disease
Source: PLoS One. 2017 Sep 8;12(9):e0184248. doi: 10.1371/journal.pone.0184248 (PMC5590908; doi:10.1371/journal.pone.0184248)
Supplement: S2 Table — The results for the categorical variables are presented in percentages in round brackets for the three genotype groups. For the normality test of the continuous variables, the Shapiro-Wilk test was used. The continuous variables were found to be non-normally distributed and described by median and interquartile range (IQR) in squared brackets. The difference between the groups was tested by Fisher’s exact test for the categorical variables and by the Kruskal-Wallis rank sum test for the interval scale measurements. A P-value of < 0.05 was considered as statistically significant. *Variables (diameter of coronary artery at worst and neutrophil %) tested for statistical significance by linear regression with age as a covariate. Significant P-values (P <0.05) are shown in bold. (DOCX) [file pone.0184248.s002.docx]

**S2 Table. Clinical characteristics of patients with KD by gender and rs1801274 (*FCGR2A*, risk allele: A) genotypes**

| Clinical variables | Clinical subgroups | Genotypes in Male (n=550) | | | |  | Genotypes in Female (n=365) | | | |
| --- | --- | --- | --- | --- | --- | --- | --- | --- | --- | --- |
|  |  | AA (n=365) | AG (n=166) | GG (n=19) | *P* |  | AA (n=221) | AG (n=132) | GG (n=12) | *P* |
| Age (year) |  | **2.5 [1.1; 3.8]** | **2.6 [1.5; 4.0]** | **3.9 [2.2; 5.8]** | **0.013** |  | 2.6 [1.4; 4.0] | 2.8 [1.5; 3.9] | 3.2 [1.8; 5.2] | 0.636 |
|  |  |  |  |  |  |  |  |  |  |  |
| Family history | No | 355 (98.1%) | 163 (98.8%) | 18 (94.7%) | 0.445 |  | 218 (98.6%) | 131 (99.2%) | 12 (100.0%) | 0.814 |
|  | Yes | 7 (1.9%) | 2 (1.2%) | 1 (5.3%) |  |  | 3 (1.4%) | 1 (0.8%) | 0 (0.0%) |  |
|  |  |  |  |  |  |  |  |  |  |  |
| Recurrence | No | 341 (94.2%) | 163 (98.2%) | 19 (100.0%) | 0.073 |  | 211 (95.5%) | 125 (94.7%) | 12 (100.0%) | 0.698 |
|  | Yes | 21 (5.8%) | 3 (1.8%) | 0 (0.0%) |  |  | 10 (4.5%) | 7 (5.3%) | 0 (0.0%) |  |
|  |  |  |  |  |  |  |  |  |  |  |
| IVIG response | Yes | 297 (83.4%) | 137 (85.1%) | 16 (84.2%) | 0.892 |  | 195 (89.9%) | 117 (89.3%) | 11 (91.7%) | 0.962 |
|  | No | 59 (16.6%) | 24 (14.9%) | 3 (15.8%) |  |  | 22 (10.1%) | 14 (10.7%) | 1 (8.3%) |  |
|  |  |  |  |  |  |  |  |  |  |  |
| CAL | CAL | 76 (20.8%) | 36 (21.7%) | 4 (21.1%) | 0.975 |  | 32 (14.5%) | 16 (12.1%) | 2 (16.7%) | 0.786 |
|  | Normal | 289 (79.2%) | 130 (78.3%) | 15 (78.9%) |  |  | 189 (85.5%) | 116 (87.9%) | 10 (83.3%) |  |
|  |  |  |  |  |  |  |  |  |  |  |
| Fever days |  | 6.0 [5.0; 7.0] | 6.0 [5.0; 7.0] | 6.0 [5.0; 7.0] | 0.307 |  | 6.0 [5.0; 7.0] | 6.0 [5.0; 7.0] | 6.5 [5.5; 7.5] | 0.342 |
|  |  |  |  |  |  |  |  |  |  |  |
| Diameter of Coronary artery at worst (mm) |  | 2.5 [2.0; 3.1] | 2.7 [2.1; 3.3] | 3.3 [2.7; 3.8] | 0.281* |  | 2.3 [1.9; 2.9] | 2.3 [2.0; 2.8] | 2.5 [2.1; 3.3] | 0.835* |
|  |  |  |  |  |  |  |  |  |  |  |
| Erythema at BCG inoculation site | No | 285 (78.1%) | 126 (75.9%) | 16 (84.2%) | 0.670 |  | 181 (81.9%) | 104 (78.8%) | 9 (75.0%) | 0.686 |
|  | Yes | 80 (21.9%) | 40 (24.1%) | 3 (15.8%) |  |  | 40 (18.1%) | 28 (21.2%) | 3 (25.0%) |  |
|  |  |  |  |  |  |  |  |  |  |  |
| CRP (mg/L) |  | 7.5 [ 3.9;11.3] | 6.8 [ 3.4;11.3] | 8.2 [ 6.7;12.6] | 0.590 |  | 7.6 [ 4.1;12.8] | 7.2 [ 3.7;11.1] | 6.8 [ 2.8;12.8] | 0.469 |
|  |  |  |  |  |  |  |  |  |  |  |
| ESR (mm/hr) |  | 57.0 36.0;7s9.0] | 58.0 [41.0;81.0] | 54.0 [37.0;86.0] | 0.584 |  | 58.0 [41.5;82.0] | 57.0 [37.0;79.0] | 71.5 [49.0;90.5] | 0.320 |
|  |  |  |  |  |  |  |  |  |  |  |
| WBC (10^9^/L) |  | 13.7 [10.8;16.7] | 13.3 [10.7;16.7] | 12.9 [ 9.4;14.6] | 0.478 |  | 13.6 [10.8;17.1] | 13.9 [11.4;17.1] | 14.2 [ 9.9;19.2] | 0.977 |
|  |  |  |  |  |  |  |  |  |  |  |
| Neutrophil (%) |  | 65.3 [53.0;75.5] | 68.0 [56.9;79.4] | 72.2 [57.3;82.5] | 0.265* |  | 68.0 [57.1;78.0] | 65.8 [55.8;76.4] | 75.4 [36.2;78.2] | 0.133* |
|  |  |  |  |  |  |  |  |  |  |  |
| Platelet (10^9^/L) |  | 327.0 [281.0;411.0] | 323.0 [271.0;393.0] | 343.0 [256.0;378.5] | 0.340 |  | 327.0 [275.0;381.0] | 334.5 [273.5;402.5] | 318.0 [213.5;406.0] | 0.573 |
|  |  |  |  |  |  |  |  |  |  |  |
| Hb (g/L) |  | 11.4 [10.8;12.1] | 11.5 [11.0;12.1] | 11.9 [10.7;12.3] | 0.140 |  | 11.5 [10.9;12.1] | 11.4 [10.9;12.1] | 11.8 [10.9;12.0] | 0.977 |
|  |  |  |  |  |  |  |  |  |  |  |
| Albumin (mg/dL) |  | 3.9 [ 3.5; 4.2] | 3.8 [ 3.5; 4.1] | 3.8 [ 3.5; 4.0] | 0.634 |  | 3.9 [ 3.6; 4.1] | 3.9 [ 3.6; 4.2] | 3.7 [ 3.5; 3.9] | 0.177 |
|  |  |  |  |  |  |  |  |  |  |  |
| AST (IU/L) |  | 34.0 [26.0;64.5] | 40.0 [26.0;95.0] | 34.0 [25.0;68.5] | 0.170 |  | 33.0 [25.0;72.0] | 32.0 [24.5;73.0] | 47.0 [24.0;132.0] | 0.860 |
|  |  |  |  |  |  |  |  |  |  |  |
| ALT (IU/L) |  | 28.0 [14.0;102.5] | 40.0 [18.0;151.0] | 67.0 [17.0;118.5] | 0.072 |  | 28.0 [15.0;117.0] | 25.0 [14.0;95.0] | 39.5 [17.5;217.0] | 0.731 |
|  |  |  |  |  |  |  |  |  |  |  |
| Total Protein |  | 6.6 [ 6.3; 7.0] | 6.5 [ 6.2; 7.0] | 6.6 [ 6.4; 7.0] | 0.778 |  | 6.7 [ 6.3; 7.0] | 6.7 [ 6.4; 7.1] | 6.7 [ 6.2; 7.3] | 0.735 |

The results for the categorical variables are presented in percentages in round brackets for the three genotype groups. For the normality test of the continuous variables, the Shapiro-Wilk test was used. The continuous variables were found to be non-normally distributed and described by median and interquartile range (IQR) in squared brackets. The difference between the groups was tested by Fisher’s exact test for the categorical variables and by the Kruskal-Wallis rank sum test for the interval scale measurements. A *P*-value of < 0.05 was considered as statistically significant.

*Variables (diameter of coronary artery at worst and neutrophil %) tested for statistical significance by linear regression with age as a covariate.

Significant *P*-values (*P* <0.05) are shown in bold.

KD, Kawasaki disease; IVIG, intravenous immunoglobulin; CAL, coronary artery lesion; BCG, Bacillus Calmette–Guérin; CRP, C-reactive protein; ESR, erythrocyte sedimentation rate; WBC, white blood cell; Hb, hemoglobin; AST, aspartate transaminase; ALT, alanine transaminase.
