# Supplementary material for: Male-specific association of the FCGR2A His167Arg polymorphism with Kawasaki disease
Source: PLoS One. 2017 Sep 8;12(9):e0184248. doi: 10.1371/journal.pone.0184248 (PMC5590908; doi:10.1371/journal.pone.0184248)
Supplement: S3 Table — A meta-analysis was performed using 3 collections including Korea GWAS, Korea replication and Japan replication data. These P-values are for the allelic model. Pooled ORs were calculated by the fixed or random-effect model. The Cochran’s Q-statistic (Phet) and I2 test were used to evaluate the between-study heterogeneity. If a Q-test showed a Phet < 0.05 or an I2 test exhibited > 50%, each of which indicates significant heterogeneity (marked in bold). (DOCX) [file pone.0184248.s003.docx]

**S3 Table. Between-study heterogeneity in a meta-analysis of associations of rs1801274 in *FCGR2A*, rs12516652 in *SEMA6A*, and rs5771303 near *IL17REL* with KD**

| SNP | Cytomap | Locus | Risk  allele | Subgroup | No.  (Cases/Controls) | Fixed effect | |  | Random effect | |  | Between–study heterogeneity | |
| --- | --- | --- | --- | --- | --- | --- | --- | --- | --- | --- | --- | --- | --- |
|  |  |  |  |  |  | OR (95% CI) | *P* |  | OR (95% CI) | *P* |  | I^2^ (%) | *P*_het_ |
| rs1801274 | 1q23 | *FCGR2A* | A | Male | 870/2,267 | 1.48 (1.28−1.71) | 1.43 × 10^-7^ |  | 1.52 (1.24−1.86) | 5.09 × 10^-5^ |  | 43.4 | 0.171 |
|  |  |  |  | Female | 591/3,035 | 1.17 (1.00−1.37) | 0.549 |  | 1.17 (1.00−1.37) | 0.549 |  | 0 | 0.911 |
|  |  |  |  | Overall | 1,461/5,302 | 1.34 (1.20−1.49) | 7.86 × 10^-8^ |  | 1.37 (1.18−1.59) | 2.54 × 10^-5^ |  | 40.2 | 0.188 |
|  |  |  |  |  |  |  |  |  |  |  |  |  |  |
| rs12516652 | 5q23 | *SEMA6A* | A | Male | 870/2,267 | 1.74 (1.28−2.36) | 3.79 × 10^-4^ |  | 1.75 (1.25−2.46) | 1.23 × 10^-3^ |  | 15.6 | 0.306 |
|  |  |  |  | Female | 591/3,035 | 1.01 (0.62−1.65) | 0.968 |  | 0.83 (0.43−1.6) | 0.5781 |  | **56.2** | 0.102 |
|  |  |  |  | Overall | 1,461/5,302 | 1.31 (1.04−1.64) | 0.020 |  | 1.30 (0.98−1.72) | 0.064 |  | 26.1 | 0.259 |
|  |  |  |  |  |  |  |  |  |  |  |  |  |  |
| rs5771303 | 22q13 | *IL17REL* | C | Male | 870/2,267 | 1.29 (1.10−1.52) | 2.10 × 10^-3^ |  | 1.34 (0.96−1.87) | 0.085 |  | **74.5** | **0.020** |
|  |  |  |  | Female | 591/3,035 | 1.15 (0.96−1.38) | 0.129 |  | 1.15 (0.96−1.38) | 0.129 |  | 0 | 0.420 |
|  |  |  |  | Overall | 1,461/5,302 | 1.22 (1.08−1.37) | 1.09 × 10^-3^ |  | 1.24 (1.06−1.46) | 8.90 × 10^-3^ |  | 39.1 | 0.194 |

A meta-analysis was performed using 3 collections including Korea GWAS, Korea replication and Japan replication data.

These *P*-values are for the allelic model.

Pooled ORs were calculated by the fixed or random-effect model.

The Cochran’s Q-statistic (*P*_het_) and I^2^ test were used to evaluate the between-study heterogeneity.

If a *Q*-test showed a *P*_het_ < 0.05 or an I*^2^* test exhibited > 50%, each of which indicates significant heterogeneity (marked in bold).

KD, Kawasaki disease; SNP, single nucleotide polymorphism; OR, odds ratio; 95% CI, 95% confidence interval.
